# Me-Doped Ti–Me Intermetallic Thin Films Used for Dry Biopotential Electrodes: A Comparative Case Study

**DOI:** 10.3390/s21238143

**Published:** 2021-12-06

**Authors:** Cláudia Lopes, Patrique Fiedler, Marco Sampaio Rodrigues, Joel Borges, Maurizio Bertollo, Eduardo Alves, Nuno Pessoa Barradas, Silvia Comani, Jens Haueisen, Filipe Vaz

**Affiliations:** 1 Centro de Física das Universidades do Minho e Porto, Universidade do Minho, Campus de Gualtar, 4710-057 Braga, Portugal; marcopsr@gmail.com (M.S.R.); joelborges@fisica.uminho.pt (J.B.); fvaz@fisica.uminho.pt (F.V.); 2 Institute of Biomedical Engineering and Informatics, Technische Universität Ilmenau, 98693 Ilmenau, Germany; jens.haueisen@tu-ilmenau.de; 3 Behavioral Imaging and Neural Dynamics Center, University “G. d’Annunzio” of Chieti-Pescara, 66100 Chieti, Italy; maurizio.bertollo@unich.it (M.B.); silvia.comani@unich.it (S.C.); 4 Department of Medicine and Aging Sciences, University “G. d’Annunzio” of Chieti-Pescara, 66100 Chieti, Italy; 5 Instituto de Plasmas e Fusão Nuclear, Instituto Superior Técnico, Universidade de Lisboa, Av. Rovisco Pais 1, 1049-001 Lisboa, Portugal; ealves@ctn.tecnico.ulisboa.pt; 6 Centro de Ciências e Tecnologias Nucleares, Instituto Superior Técnico, Universidade de Lisboa EN10, 2695-066 Bobadela, Portugal; nunoni@ctn.tecnico.ulisboa.pt; 7 Department of Neuroscience Imaging and Clinical Sciences, University “G. d’Annunzio” of Chieti-Pescara, 66100 Chieti, Italy; 8 Department of Neurology, Biomagnetic Center, Jena University Hospital, 07747 Jena, Germany

**Keywords:** thin-films, binary intermetallic systems, Ti–Me dry electrodes, polymers, electrode–skin impedance, biopotential measurement

## Abstract

In a new era for digital health, dry electrodes for biopotential measurement enable the monitoring of essential vital functions outside of specialized healthcare centers. In this paper, a new type of nanostructured titanium-based thin film is proposed, revealing improved biopotential sensing performance and overcoming several of the limitations of conventional gel-based electrodes such as reusability, durability, biocompatibility, and comfort. The thin films were deposited on stainless steel (SS) discs and polyurethane (PU) substrates to be used as dry electrodes, for non-invasive monitoring of body surface biopotentials. Four different Ti–Me (Me = Al, Cu, Ag, or Au) metallic binary systems were prepared by magnetron sputtering. The morphology of the resulting Ti–Me systems was found to be dependent on the chemical composition of the films, specifically on the type and amount of Me. The existence of crystalline intermetallic phases or glassy amorphous structures also revealed a strong influence on the morphological features developed by the different systems. The electrodes were tested in an in-vivo study on 20 volunteers during sports activity, allowing study of the application-specific characteristics of the dry electrodes, based on Ti–Me intermetallic thin films, and evaluation of the impact of the electrode–skin impedance on biopotential sensing. The electrode–skin impedance results support the reusability and the high degree of reliability of the Ti–Me dry electrodes. The Ti–Al films revealed the least performance as biopotential electrodes, while the Ti–Au system provided excellent results very close to the Ag/AgCl reference electrodes.

## 1. Introduction

Commonly used conventional electrodes for biopotential recordings, in clinical and research applications, are silver–silver chloride (Ag/AgCl) based electrodes, in combination with electrolyte gels or pastes. These electrodes present the characteristics of non-polarizable electrodes including outstanding reliability, and low, almost frequency-independent skin contact impedance. Thus, Ag/AgCl electrodes are considered the gold standard for measurements involving low voltage signals of electrophysiological origin such as e.g., electromyography (EMG), electroencephalography (EEG), or electrocardiography (ECG).

Nevertheless, and despite their popularity, the gel-based electrodes present several considerable drawbacks such as short service life, extensive preparation time, and skin reaction ranging from liquid-related discomfort to eventual allergic contact dermatitis [1,2]. Furthermore, the use of gel has the further drawback of dehydration, leading to increased skin contact impedance and motion artifacts, in turn reducing the signal quality and limiting the electrode application environment and lifetime [3,4].

To overcome the limitations of conventional gel-based electrodes, improved hydrogels [2,5,6,7] and semi-dry [8,9,10,11] and dry electrodes [3,12,13,14] have been proposed and increasingly used in recent years. Semi-dry electrodes have been suggested as a compromise between conventional gel-based and completely dry electrodes. By applying a small amount of electrolyte to the electrode–skin point of contact, a stable, low-impedance contact can be established, without individual electrode preparation and without major gel residues in the hair. However, semi-dry electrodes are primarily used for EEG applications and are subject to an inherent limitation of the electrolyte in the respective reservoir. Consequently, similar to conventional gel-based electrodes they are not suitable for long-term applications. Furthermore, the advantages of using semi-dry electrodes for applications on bare skin and skin with low hair density in ECG and EMG are limited and do not outweigh the increased requirements for preparation, cleaning, and disinfection. On bare skin, dry electrodes normally exhibit higher contact impedances than the conventional gel-based wet electrodes, but when combined with state-of-the-art electronics, following a short settling time, the electrodes can record bioelectric signals with signal-to-noise ratios similar to the gel electrodes. Moreover, the use of soft, flexible substrate materials fosters the adaptivity of the electrode to the body shape, increasing the long-term wearing comfort and improving the electrode–skin contact, especially during body motion [3,12,13,14]. Dry electrodes are thus user-friendly solutions offering improved applicability without the need for hydrogels or other types of wet electrolytes [3,12,13,14,15,16,17].

Different dry electrode shapes, grades of flexibility, and material compositions have been suggested, depending on the intended application (e.g., EMG, ECG, or EEG) and respectively required properties. The current state-of-the-art in dry and semi-dry electrodes is the use of flexible materials, either coated or doped with electrically conductive components. The range of doped materials primarily includes elastomers doped with graphite [18,19], and silver (Ag) [20,21] components, as well as carbon nanotubes and nanofibers [19,22,23]. Production cost, complexity, and reproducibility as well as constraints in electrode shape, mechanical characteristics, and durability have limited these materials to prototypes thus far. Coated elastomers have been suggested primarily with Ag [24], Ag/AgCl [11,13,16,25], gold (Au) [26], and titanium (Ti) [17,27,28]. While silver-based coatings are known to provide excellent electrochemical and thus bioelectric signal characteristics, their biocompatibility in long-term repetitive applications remains an often discussed and questioned limitation [29]. The costs of gold coatings have also limited these electrode types from widespread use. Ti-based coatings have been proposed as pure metal coatings and in various compositions. A major advantage of these coatings is their well-known biocompatibility and a wide range of established medical applications [30,31]. Moreover, thin-film technology applied to common flexible polymer substrates presents important alternatives aimed at cost-efficient, flexible, and reliable biopotential dry electrodes solutions. The possibility to customize the films’ nanostructures profiting from the low weight and flexibility of the polymer substrates paves the way for the development of a new generation of biomedical sensors. The use of biocompatible thin films enables the activation and functionalization of non-conductive surfaces. When deposited on mechanically flexible polymers, the thin film provides highly conductive pathways for biosignals without compromising the polymer’s elastic modulus, while improving the surface mechanical resistance [17,32]. However, a systematic comparison of Ti-based thin film compositions used for bioelectric signal acquisition is missing to date, since existing literature focuses on individual metal combinations only and lacks assessment of cross-system differences.

Our investigations focused on a multi-parameter study and comparison of the most promising Ti-based coatings, dedicated to the development of dry electrodes for biopotential measurement. We functionalize flexible polyurethane substrates with different Ti–Me binary intermetallic thin films doped with increasing amounts of Me metals. The Ti–Me intermetallic thin films were prepared by magnetron sputtering, an efficient, economic, and environmentally friendly technology, chosen for its versatility and high adaptability to industrial processes. Ti is a biocompatible material and Ti-based alloys are being applied in an increasing number of medical applications due to their excellent mechanical, physical, and biological performance. By adding a metal “Me” to the Ti matrix, we aimed to improve the well-known properties of pure Ti due to the formation of intermetallic-like compounds. Without considerable differences in biocompatibility and considering their applicability in biomedical sensing, the functional properties of intermetallic compounds proved to be far more advantageous than those of pure metals [27,30,32,33,34,35]. In the binary Ti–Me intermetallic thin films systems the electrical, chemical, and mechanical characteristics of the individual elements prevail [27,32,33]. The Ti–Me dry electrodes were assessed in terms of electrode–skin impedances, which are an important parameter of electrode–skin contact.

Electrode–skin interfacial impedance spectroscopy was performed in vivo on 20 young volunteers during sports activity, comparing Ti–Me thin films deposited on both PU and SS substrates with conventional hydrogel-based adhesive electrodes. Furthermore, the same pair of Ti–Me dry electrodes was used over all the in-vivo tests (20 measurements including cleaning and disinfection over the course of 14 days) providing a qualitative measure of the electrodes’ stability and wearing effects on the thin film.

## 2. Materials and Methods

### 2.1. Ti–Me Dry Electrode Preparation

The dry electrodes were prepared by deposition of Ti–Me thin films on flexible polyurethane, PU, substrates and stainless steel, SS, discs, using a custom-made sputtering deposition system. For reference and comparison purposes, commercial hydrogel-based Ag/AgCl electrodes (SNAP Euro ECG electrodes, Foam-Solid gel, FIAB, Firenze, Italy) with a base diameter of 15 mm, were also used.

**Figure 1:**
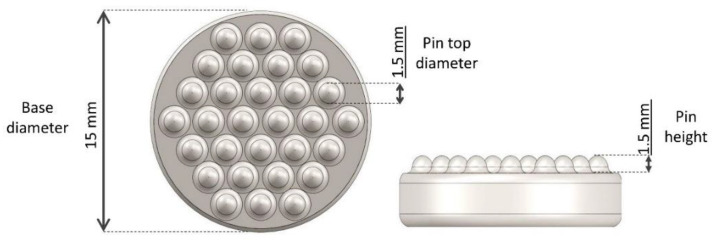
Top and side-view of the multiwave PU substrate containing 30 wave pins in a honeycomb configuration. The relatively short waves provide an improved feeling of comfort and ensure proper interfacial electrode–skin contact [15,37].

The PU substrates (Biresin U1419, Sika, Germany) adopt a multiwave design previously validated for EEG applications [15,36]. The multiwave electrode concept, as well as the wave height and arrangement (see Figure 1 for details), was implemented to maximize the ability of the electrodes to pass through an eventual minor hair layer on the forehead during EEG measurements [15,37]. The same design was adopted for this study. The selective deformation of the PU waves allows them to easily pass through hair on the skin, while the adaptivity to irregular body contours is provided by the PU flexibility, avoiding the unpleasant procedure of shaving the skin before a surface EMG (sEMG) recording when using flat (disc) electrodes.

The use of SS enabled the investigation of the substrate influence on the overall behavior and stability of the Ti–Me electrodes. The SS discs (reference AISI 316 L, Roqlaser, Portugal) were produced with a thickness of 0.5 mm, while the base diameter (15 mm) was kept equal to the polymer substrates. In addition, silicon substrates (~400 mm^2^) with (100) orientation (boron-doped p-type) were added to the sputtering chamber during the Ti–Me thin film depositions for subsequent chemical and microstructural characterization analysis.

Prior to the depositions, the substrates were cleaned with ethanol and activated by plasma treatment in pure Argon (Ar) atmosphere (working pressure of 80 Pa) to ensure strong adhesion of the Ti–Me films to the substrates. The plasma treatments were promoted during 900 s by a plasma cleaner (Plasma System Zepto, Diener electronic GmbH & Co. KG, Ebhausen, Germany), equipped with a 13.56 MHz generator, at a power of 50 W and a rotary pump [16,38]. This procedure was especially important to increase the surface energy and wettability of the PU substrates without modifying the structural properties of the bulk polymer [39,40,41].

After the activation, the substrates were placed on a 3-dimensional grounded sample holder positioned in the center of the sputtering chamber at 70 mm from the magnetron. For the depositions, a Ti target (purity: 99.99 at.%, dimensions: 200 mm × 100 mm × 6 mm) was modified with metallic pellets of Al, Cu, Ag or Au (area: 16 mm^2^, thickness: 0.5 mm) homogeneously glued with conductive Ag glue over the racetrack of the target. The type and number of Me pellets varied according to the Ti–Me system under preparation in order to achieve a wide range of chemical compositions for each system. The depositions were carried out at room temperature, keeping the Ar flow constant while the substrate holder was in a rotation mode (speed of 5 rpm) to ensure the homogeneity of the deposited samples. The direct current (DC) density was set to 75 A/m^2^ and the maximum deposition temperature reached was 60 °C, avoiding thermal degradation of the PU substrates [39]. The base pressure was always below 2.0 × 10^−4^ Pa, and the Ar pressure was kept constant at 3.0 × 10^−1^ Pa.

### 2.2. Chemical and (Micro)Structural Analysis

The quantitative chemical composition of the Ti–Me thin films was evaluated by Rutherford backscattering spectrometry (RBS) analysis. The analysis was carried out in a small RBS chamber, using monoenergetic and collimated beams of 4 He^+^ (1.5–2 MeV) and/or 1 H^+^ (2.3 MeV) ions accelerated ~2.5 MeV by a Van der Graaf accelerator, to perpendicularly collide on the sample. Inside the chamber, three detectors were positioned to record the energies of the backscattered ions: one Si surface barrier detector located at a 140° scattering angle, and two pin-diode detectors located symmetrical to each other, both at 165° (detector 3 on the same side as detector 2). The angle of incidence was 0° (normal incidence). The in-depth composition profiles were simulated using the NDF software, after three different measurements in each sample [42,43].

The crystal structure and phase distribution of the Ti–Me thin films were carried out employing X-ray diffraction (XRD), using a Bruker D8 Discover diffractometer, operating with Cu–Kα radiation (λ = 1.5406 Å). The XRD patterns were deconvoluted, assuming the Rietveld method with a Pearson VII function. The corresponding structures were analyzed by means of the Inorganic Crystal Structure Database (ICSD) using the TOPAS software (Bruker Corp., Billerica, MA, USA).

The plan-view and cross-section micrographs used to evaluate the morphological features and to determine the thickness of the Ti–Me thin films were performed using a high-resolution scanning electron microscope (SEM; FEI Nova NanoSEM 200), with X-ray microanalysis and electron backscattered diffraction analysis, operating at 15 keV.

### 2.3. In-Vivo Electrode–Skin Interfacial Impedance Measurements

All electrode types were tested on a group of 20 healthy male volunteers, age 23.7 ± 3.3 years, regular practitioners of cycling at least twice a week. The participants reported no recent history of physical injuries, chronic diseases (e.g., neurological or dermatological diseases) or use of drugs. The study was approved by the Ethics Committee of the University “G. d’Annunzio” of Chieti-Pescara (Italy) and all volunteers provided written informed consent.

Each volunteer wore two pairs of electrodes of the same type on the (i) rectus femoris and (ii) vastus medialis of the dominant leg. The electrodes were placed on the skin after cleaning with 70% ethyl alcohol and with a distance of 20 mm center to center in the direction of the muscle fibers, following the Surface ElectroMyoGraphy for the Non-Invasive Assessment of Muscles (SENIAM) guidelines [44]. Rigid zinc oxide strapping tape (Strappal, BSN Medical, Hamburg, Germany) was used to fix and keep the same pressure applied to all the electrodes.

**Figure 2:**
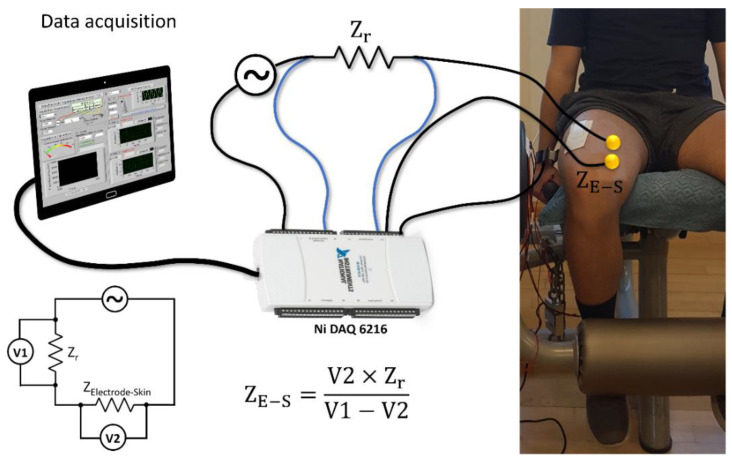
Measurement setup for the electrode–skin interfacial impedance using a custom-made impedance measurement device in a two-pole setup on the muscle of the subject’s dominant leg.

The electrode–skin interfacial impedance was then measured with a custom-made and portable device constituted by a USB-6216 Isolated Multifunction I/O data logger (National Instruments, Austin, TX, USA) in combination with a custom-made LabView software. Participants were asked to stay relaxed and quiet during the measurements. The safety of the volunteers was ensured by the design of the measurement setup and circuitry, cutting off eventual electric currents exceeding the maximum allowed value of 50 μA [17,45]. The absolute electrode–skin interfacial impedance value (Z_E-S_) was determined by using a voltage divider circuit. A known voltage V1 was applied to measure V2, using a known impedance value (Z_ref_), as schematically depicted in Figure 2.

This two-pole setup provided a measure of the overall impedance of both the electrode–skin interface and the biological tissue between the electrodes. Nevertheless, due to the methodical and reproducible experimental procedure on the electrodes’ placement, the tissue impedance was considered as not relevant for the quantitative comparison of the Ti–Me electrodes. Prior to the measurements, the custom-made portable impedance measurement device was calibrated using a commercial impedance analyzer (4192A LF, Hewlett Packard Company, Palo Alto, CA, USA) in a standard two-port setup. For the impedance range of interest, we received an error of 0.15% [17,37].

The electrode–skin interfacial impedance was determined for five distinct frequencies: 10 Hz, 50 Hz, 100 Hz, 500 Hz, and 1000 Hz, after a 60 s stabilization period, in potentiostatic measurement mode. The overall duration of each impedance spectroscopy per volunteer was about 5 min. Given the foreseen use of the Ti–Me thin films in the biomedical field as dry biopotential electrodes, the selected frequency values covered the frequency range of the standard biopotential signals of EEG (1–40 Hz [4]), sEMG (6–500 Hz [46]), and ECG (0.05 Hz–150 Hz up to 700 Hz [47]) [48].

The sequence of tests for all Ti–Me electrodes (PU and SS substrates) and the Ag/AgCl reference electrodes was randomized to avoid eventual systematic influences on the results. The same pair of Ti–Me dry electrodes was used for the 20 volunteers. After each application, the dry electrodes were removed, cleaned, and disinfected with 70% ethyl alcohol. Contrarily, the self-adhesive hydrogel-based Ag/AgCl reference electrodes were disposed of after each test.

## 3. Results

### 3.1. Ti–Me Systems—Chemical Composition

**Figure 3:**
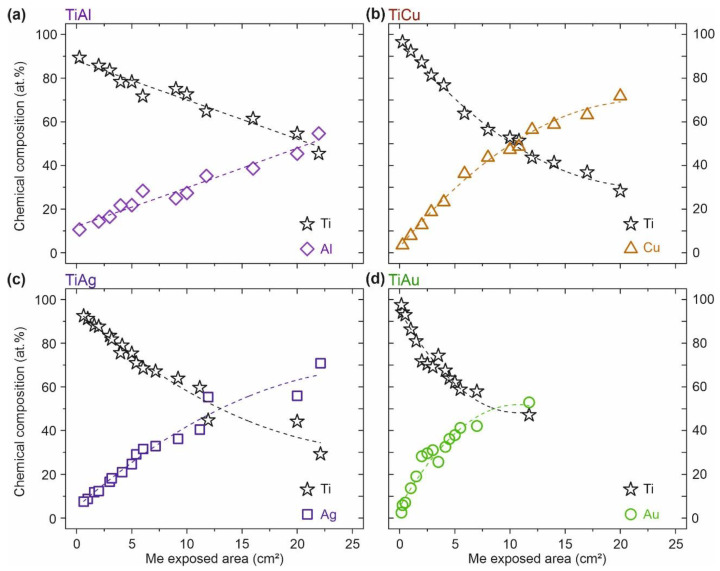
Evolution of the atomic composition (at.%) of the four Ti–Me intermetallic systems obtained by RBS spectra analysis: (**a**) Ti–Al, (**b**) Ti–Cu, (**c**) Ti–Ag, and (**d**) Ti–Au. The connecting dashed lines represent the composition evolution trend of each system.

The chemical composition of the four different Ti–Me intermetallic systems obtained by RBS spectra analysis, as a function of the Me exposed area on the target, is shown in Figure 3. The error in the atomic concentration determination was about 3 at.% for Al and around 0.5 at.%. for the other chemical elements (Ti, Cu, Ag, and Au).

All the Ti–Me systems were deposited with Me areas ranging between 0.16 cm^2^ and 22 cm^2^, with exception of the Ti–Au system, where the maximum Au exposed area was about 12 cm^2^, for purely economic reasons. As expected, the results evidence that the Ti and Me content in the films varied in inverse proportion with the Me exposed area on the target. However, a closer look at the results (Figure 3) reveals the great influence of the sputtered metal Me on the chemical composition of the films. For instance, the Ti content in the Ti–Me thin films decreased to 50 at.% for exposed areas of Cu and Ag (~12 cm^2^) (Figure 3b,c), whereas in the Ti–Al system it was necessary to expose about 21 cm^2^ of Al pellets to have the same Ti content value (Figure 3a). By contrast, with only 6 cm^2^ of Au pellets in the racetrack of the Ti target, it was possible to obtain a 50:50 concentration thin film of Ti–Au (Figure 3d). All prepared Ti–Me films proved to be chemically homogeneous in depth, with no evidence of selective diffusion, regardless the number of Me pellets placed onto the Ti target [32]. Since the atomic concentrations of Ti and Me showed an opposite trend, the Me/Ti atomic ratio was be adopted from then on to correctly identify the samples, providing a simple way to establish a comparative analysis between the systems.

The wide range of chemical compositions in which the four different Ti–Me systems were prepared gave rise to the development of a unique set of structural and morphological characteristics. In the same way, the impedance of the electrode on the skin would have been a direct result of its physical response, which is a consequence of the exclusive (micro)structural features that each thin film developed.

### 3.2. Ti–Me Systems—Structural Composition

The addition of aluminum, copper, silver, or gold to an initial matrix of Ti opened a wide range of structural arrangements. Each Ti–Me system had developed unique structural features exclusively depending on the Me dopant and its concentration in the film. Three main structural zones were identified to be common to all the Ti–Me systems prepared: (i) a Ti-rich zone, representing all the films dominantly composed by Ti; (ii) an intermetallic zone, for films where the precipitation of Ti–Me intermetallic phases became evident; and (iii) a Me-rich zone when the metal, Me, plays the major role in the films’ composition.

**Figure 4:**
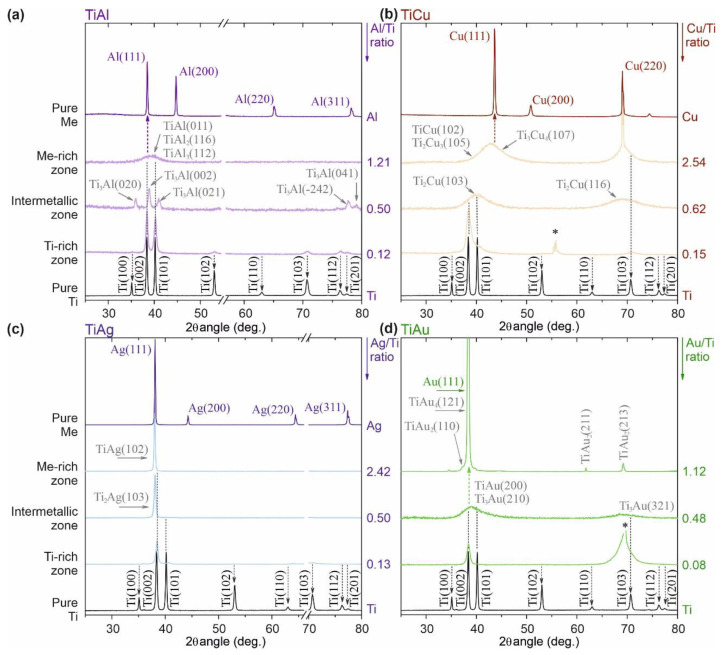
X-ray diffraction patterns of the Ti–Me representative films attending to the Me/Ti ratio for every system prepared: (**a**) Ti–Al system, (**b**) Ti–Cu system, (**c**) Ti–Ag system, and (**d**) Ti–Au system. For Ti–Me systems prepared with Al (a) and Ag (d), a break in the scale of the diffraction angle (*x*-axis) was introduced, to highlight the main crystallographic orientation growths of both systems.

To simplify the structural analysis, representative XRD diffraction patterns of each Ti–Me system were selected and are depicted in Figure 4. For every system, five XRD diffraction patterns are presented, including the reference samples of pure Ti and Me thin films except for the pure Au (for the previously stated economic reasons).

In the Ti-rich zone, all the Ti–Me films showed diffraction patterns very similar to the polycrystalline Ti thin film that crystallizes into a high close-packed hexagonal (hcp) structure (ICSD collection code #44872). Different crystallite orientations can be observed, with (002) being the preferred, as foreseen by Petrov et al. for hcp structures [49]. However, the incorporation of the Me atoms into the Ti crystal lattice (in substitutional or interstitial sites), even in low contents (Me < 15 at.%) leads to less intense and broader diffraction peaks. Thus, and despite being representative of the Ti hcp structure, the addition of an alloying element (Me) into the Ti matrix promotes considerable changes in the cell parameters, which were evidenced by the suppression of some crystallographic orientations (e.g., (100) or (110)). The results suggest that all the Ti-rich films showed solid solutions characteristics (Ti = solute, Me = solvent), developing α-Ti (Me) metastable phases with cumulative local disorder and loss of crystallinity. Indeed, the low temperatures employed during the Ti–Me depositions strongly limited the atomic mobility, hindering the diffusion of species favoring the formation of metastable phases with reduced structural order [49,50,51]. The broadened and less intense Brag’s peaks/smaller crystallite sizes were evident for the Ti–Cu and Ti–Au films (Figure 4b,d) prepared in this zone. Nevertheless, and although in minor traces, evidence of the (002), (101), and (103) diffraction planes of the pure Ti film could still be noticed for the 0.12 Al/Ti and 0.13 Ag/Ti films, suggesting higher solubility of Al and Ag into the Ti matrix [32].

In the intermetallic zone, i.e., for the Ti–Me films prepared with Me/Ti ratios above 0.20, the most evident structural feature was the precipitation of the intermetallic phases in the α-Ti (Me) solid solution. However, a double trend of structural evolution for the prepared Ti–Me systems was evident.

On the one hand, the Ti binary systems of Al and Ag (Figure 4a,c) evidenced polycrystalline thin films with the emergence of a new set of diffraction peaks attributed to intermetallic phases: Ti_3_Al (ICSD collection code #191189), and Ti_2_Ag (ICSD collection code #605931), respectively [32]. However, the increasing addition of Me (Al or Ag) led to the cumulative local lattice disorder resulting in the progressive amorphization of the α-Ti structure that became less evident in the diffraction patterns.

On the other hand, the appearance of noticeable broad diffraction humps for the films prepared with Au or Cu left no doubt about the formation of non-crystalline amorphous structures typically featured on thin film metallic glasses (TFMGs), as discussed in previous studies [32]. The location of those broad diffraction patterns (Figure 4b,d) suggests the formation of different poorly-crystallized Ti–Cu (ICSD collection codes: #629388 for Ti_2_Cu and #103130 for Ti_3_Cu), and Ti–Au (ICSD collection code #58605 for Ti_3_Au or #197280 for TiAu) intermetallic metastable phases [32]. The TFMG behavior observed for these systems can be explained by the low solubility of Cu and Au into the Ti matrix [32] but also by the kinetic limitations employed within the sputtering process parameters (low temperatures and low-energy ion bombardment without biased substrates) [49,52].

In the transition to the Me-rich zone, the structural evolution trend for the systems prepared with Al and Au presented a turning point. The Al-rich films started to exhibit a quasi-amorphous structure evidenced by a broad diffraction domain (Figure 4a), where several Ti–Al intermetallic phases could be indexed (ICSD collection codes: #290974 for TiAl, #107009 for TiAl_2_ and #190891 for TiAl_3_), albeit it was impossible to distinguish them due to their reduced structural order [32]. Contrarily, the Au-rich film evolved to Au-rich crystalline domains (ICSD collection codes: #58607 for TiAu_2_, #109132 for TiAu_4_) reflected in sharp diffraction peaks with reduced full width at half maximum (FWHM), Figure 4d.

No considerable structural changes were observed in the intermetallic zone with growing Me addition for the Ti–Ag and Ti–Cu systems. In the Ag-rich films, the quasi-crystalline Ti–Ag intermetallic phase (ICSD collection code #605934) prevailed, following the behavior observed for the Au-rich films. Additionally, for Ag- and Au-rich films, the deviation towards the same angular position of the fcc–Ag and Au structures together with the unfavorable thermodynamic conditions during the films’ growth, suggested the development of silver- and gold- doped Ti, Ag (Ti), and Au (Ti), metastable phases in crystalline nanophases coexisting with the intermetallic ones [32,49]. For the Ti–Cu system, the glassy amorphous structures—typical of TFMG—remained despite the shift of the diffraction hump to higher values of 2 θ observed for the film with 2.54 Cu/Ti ratio film. In accordance with the Ti–Cu equilibrium phase diagram [53], several poorly crystallized Cu-rich metastable phases (ICSD collection codes: #103128 for TiCu, #103133 for Ti_2_Cu_3_ and #103134 for Ti_3_Cu_4_) with a great tendency to amorphization could be associated with the diffraction hump located within the range (38° to 48°).

The mutual solubility of the elements in the Ti–Me binary systems played a prominent role in the structural characteristics observed for the films prepared within the Me-rich zone. Results show that the high solubility of Ti in Ag- and Au-rich films (crystalline structures) and the low solubility in the films prepared with the highest contents of Cu and Al (amorphous structures) were most certainly playing a decisive role in the overall behavior [53,54,55,56].

### 3.3. Ti–Me Systems—Morphological Evolution

**Figure 5:**
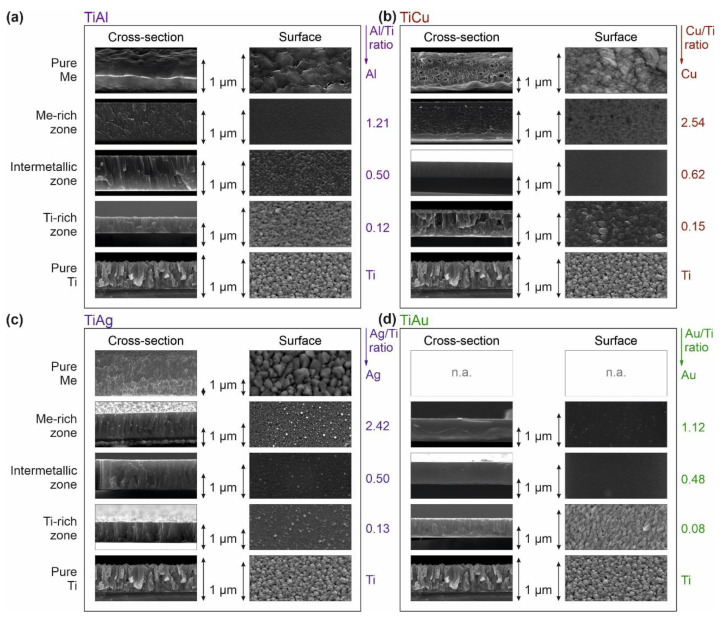
Morphology evolution of the Ti–Me films attending to the different structural zones identified: (**a**) Ti–Al system, (**b**) Ti-Cu system, (**c**) Ti-Ag system, and (**d**) Ti–Au system. The morphology of the pure Ti and Me metallic thin films are included to be used as reference.

The morphological features of the Ti–Me thin films were assessed in the light of the different structural arrangements already identified. For each system, three representative films were selected, one from each structural zone (Ti- or Me-rich zone and intermetallic zone). To simplify the comparison of the morphological evolution, additional micrographs of the pure Ti and Me thin films were included. The plan-view and cross-section micrographs of each representative film, deposited on silicon substrates, can be seen in Figure 5.

Regardless of the Ti–Me system, all the thin films prepared in the Ti-rich zone presented a columnar-like growth typical of the Ti structures, which is very common on sputter deposition processes at low temperatures and low-energy ion bombardments [57,58]. The non-thermodynamic equilibrium conditions were also responsible for the rough surface morphologies observed in this zone, which were especially evident in the Ti–Al and Ti–Au systems and reminded us of the three-dimensional hexagonal grain features of the Ti surface. Due to the sputtering process’ kinetic limitations, the atoms of Ti and of the respective metal do not arrive uniformly at the substrate (with the same energy), therefore contributing to the surface growth roughness phenomenon [59,60].

The morphology of the Ti–Me films prepared in the intermetallic zone reflected the distinct trends of the structural evolution previously discussed. The films prepared with Al and Ag continued to reveal the columnar growth typical of Ti (visible on the reference film), although developing denser and less porous microstructures (Figure 5a,c), which may have been related to the precipitation of crystallized intermetallic nanostructures. The formation of the Ti–Ag and Ti–Al intermetallic phases was also responsible for the coarsening of crystalline grains observed on the surface morphology, giving rise to rougher surfaces.

In turn, the amorphous humps discussed for the structural analysis of Ti–Cu and Ti–Au films gave rise to vitreous and amorphous microstructures with smooth and featureless surfaces (Figure 5b,d), a hallmark of TFMGs structures [61,62,63]. These denser and close-packed microstructures completely deviated from the microstructure of the Ti-reference film and could be explained by the almost absence of grain boundaries (crystallite sizes less than 2 nm).

The microstructures presented by the films prepared with higher Me contents (Me-rich zone) could easily be distinguished from the pure Me reference films prepared under the same conditions. These results evidence the wide range of morphological features that can be achieved between the metallic counterparts used as reference (Ti and Me pure thin films) by employing the sputtering process. Within this zone, the surface of Al-rich films was surprisingly smooth, which was certainly related to the substantial loss of crystallinity. The Cu-rich and Au-rich films were generally smoother, although some additional fine grain-like features could be observed on the top surface (more evident on the Ti–Au system). The Ti–Ag system deviated from the others by the formation of some spherical-like grains resembling “chip cookies”, dispersed throughout the entire microstructure and even emerging at its surface. The chemical weight contrast analysis (Figure 5c) suggested that these visible bright nanoparticles may have corresponded to Ag-rich crystalline phases, corroborating the XRD results. Regarding the films’ growth, Ti–Al and Ti–Ag systems developed full-dense microstructures with remaining traces of the columnar growth, whereas for Ti–Cu and Ti–Au systems it was possible to observe shear striation on denser and featureless microstructures. Considering the structural analysis, the partial vein pattern features revealed by the Cu-rich films can be easily associated with the fracture of metallic glassy samples, attesting once again to the TFMG behavior of the Ti–Cu system for ratios above 0.25 [64].

### 3.4. Electrode–Skin Impedance Measurements

**Figure 6:**
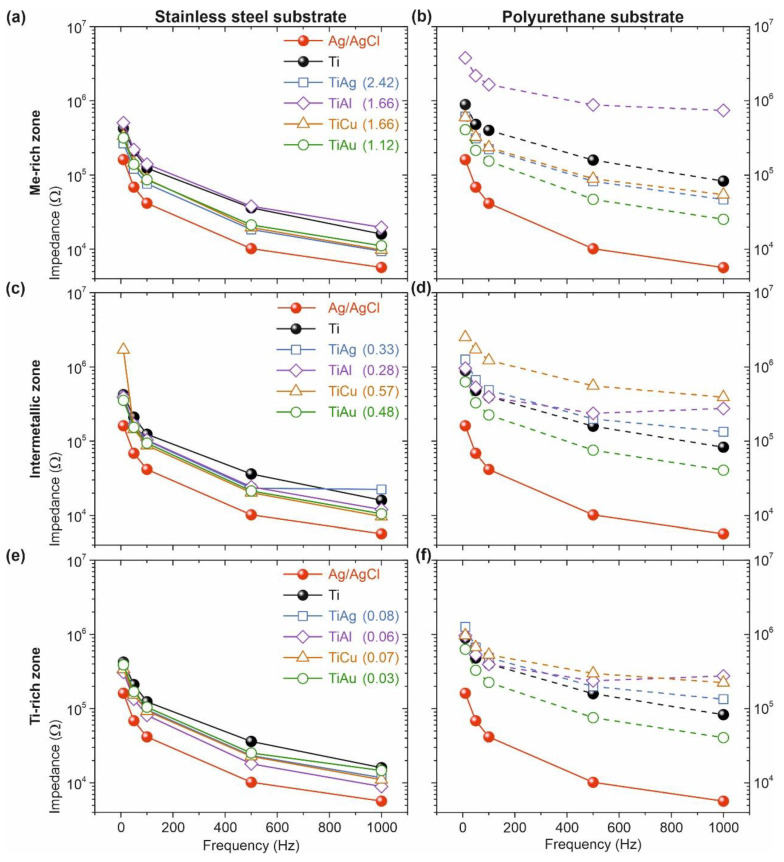
Mean values of impedance at the electrode–skin interface, over a wide range of frequencies, were measured on 20 young sportsmen volunteers. For each Ti–Me system, three representative films of the Me-rich zone (**a**,**b**), intermetallic zone (**c**,**d**), and Ti-rich zone (**e**,**f**) were tested as electrodes using both substrates. Stainless steel, SS and polyurethane, PU. The electrode–skin impedance values for the reference Ag/AgCl hydrogel electrode (red line) and the thin Ti film used as reference (dark line) are also presented.

The electrode–skin interfacial impedance study was carried out under well-defined conditions to properly investigate the use of the Ti–Me thin films as dry contact electrodes for biopotential monitoring. All the electrodes, including the reference hydrogel-based Ag/AgCl electrodes, were tested on all volunteers in randomized sequence, adopting the procedure described in Section 2.3. The obtained results, presented in Figure 6, take into consideration the substrates used and the (micro)structural zone of the prepared films.

For all tested electrodes, including the conventional hydrogel electrodes of Ag/AgCl recognized as non-polarizable in the biomedical field [37,48], the contact impedance depended on frequency. As expected, the impedance of dry electrodes based on Ti–Me thin films was always higher than the hydrogel-based electrodes [1]. The replacement of an ion-rich electrolyte (incorporated into the hydrogel Ag/AgCl reference electrodes) by a natural perspiration interface for the Ti–Me dry electrodes increased the interfacial impedance [48].

Despite the higher contact impedance values, the overall Ti–Me nanoelectrodes’ absolute impedances follow the trend exhibited by the Ag/AgCl electrodes. The higher impedance at lower frequencies, related to the capacitive components of the contact decreased at higher frequencies values [48]. Nevertheless, for the dry electrodes prepared in the Ti- and Me-rich zones on PU substrates, a partly less pronounced impedance decrease compared to the hydrogel-based reference electrodes could be observed and might be related to a change in the capacitive interface developed by these thin films [12,37,48].

One of the most interesting outcomes of the impedance measures is that pure Ti film generally exhibited the highest impedance values for the electrodes with SS substrates, showing the importance of doping the Ti matrix with Me metals. In fact, the obtained results highlight the influence of the substrate on the contact impedance of the dry electrodes. Ti–Me electrodes with SS substrates revealed impedance values at the same order of magnitude as the Ag/AgCl reference electrodes, whereas the same thin films deposited on PU substrates exhibited contact impedances up to two orders of magnitude higher than that of the hydrogel-based reference electrodes (for frequencies above 500 Hz).

The comparison of the electrode–skin contact impedance for the different Ti–Me systems underlined the influence of the Me used or the Me/Ti atomic ratio on the impedance behavior. Considering the flexible electrodes based on PU substrates, it was possible to observe that the increase of Cu or Ag content in the films was associated with important variations of the interfacial impedance. Initially, in the Ti-rich zone, a difference of two orders of magnitude was observed relative to the reference electrode. This behavior got worse in the intermetallic zone and was improving in the Me-rich zone. Here, the values approached the reference electrode and were lower than those obtained for the pure Ti film (with Ti–Al film as an exception). On the other hand, all electrodes prepared with Au presented linear and consistent electrode–skin impedances. This behavior was especially evident for the Ti–Au film prepared in the Me-rich zone, where the impedance using PU substrates was in the same order of magnitude as the electrodes with SS substrates. Conversely, the Ti–Al system deposited on PU substrates exhibited very high values of contact impedance for all tested frequencies.

**Figure 7:**
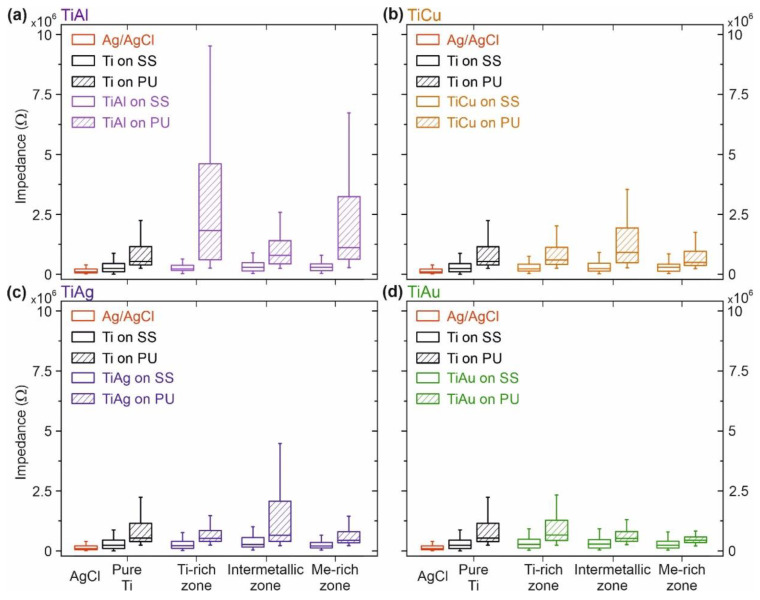
Electrode–skin impedance distributions of the Ti–Me electrodes, acquired in 20 volunteers and two skeletal muscles (rectus femoris and vastus medialis), at a frequency of 10 Hz. (**a**) Ti–Al electrodes, (**b**) Ti–Cu electrodes, (**c**) Ti–Ag electrodes, and (**d**) Ti–Au electrodes. The Ti electrode is included in the representation of each system for comparison purposes. For each volunteer, the measurements were performed using every Ti–Me condition and both substrates: stainless steel (SS) and polyurethane (PU). Each distribution (boxplot) includes data from one set of electrodes (two pairs, one for each muscle), (re-)used in all volunteers, except the case of the disposable reference electrodes of Ag/AgCl (one set per volunteer).

Notwithstanding the differences between the Ti–Me films and the conventional Ag/AgCl reference electrodes, the observed level of interfacial impedance does not necessarily affect the quality of the signals recorded with state-of-the-art biosignal amplifiers [13,37,65]. The stability of the interfacial contact and the balance between the electrode pair (reduced variability) and its reproducibility over time [66,67] are equally important aspects in the development of reliable biopotential dry electrodes. For this reason, the distribution of the impedance values for all volunteers (N = 20) using the same Ti–Me electrode pair was assessed for the lowest test frequency of 10 Hz (see Figure 7).

In comparison to conventional Ag/AgCl electrodes, the Ti–Me dry electrodes showed higher variability, especially evident for PU substrates. In opposition, no considerable differences were observed in the impedance distributions between the Ti–Me electrodes prepared with SS substrates. The larger variability of the results obtained for PU substrates could have been due to the fact that used polymers are flexible materials, meaning that the Ti–Me films deposited on such substrates were more prone to external stresses resulting from mechanical solicitations. The stretching behavior of the Ti–Ag thin films deposited on polymeric substrates has been previously studied by the authors using fragmentation tests [27]. The addition of Ag to the Ti matrix significantly improved the fracture resistance and reduced the crack density of Ti-Ag films. The Ag-rich crystalline domains developed by the Ag-rich films ensured reliable electrically conductive pathways, even after crack propagation.

The results on the impedance variation highlight the strong dependence of PU-based electrodes on the type and Me content used in the Ti–Me nanomaterials. Electrodes with Ti–Ag and Ti–Cu films produced in Ti- and Me-rich zones, exhibited slightly lower contact impedances (~7.0 × 10^5^ Ω) than those prepared in the intermetallic zone (~1.7 × 10^6^ Ω). This behavior was transversal to all the frequencies and to SS-based electrodes. The higher variability of interfacial impedance values, observed for the intermetallic zone, can be correlated with the formation of brittle Ti–Me metastable intermetallic phases, which are less likely to withstand mechanical stress. A quite different behavior was observed for the Ti–Al electrodes, that showed the highest variability of the interfacial impedances (~3.0 × 10^7^ Ω in the Ti-rich zone) with the highest medians, mainly on Ti- (~1.7 × 10^6^ Ω) and Me-rich (~1.0 × 10^6^ Ω) zones. It seems evident that Ti–Al films have major difficulties in following up the mechanical solicitations of PU substrates, when applied on the skin. This behavior might be related to the columnar morphologies, that are less resistant to plastic deformations within the Ti-rich zone, and to the severe loss of crystallinity registered for the Me-zone. In contrast, the Ti–Au electrodes based on PU substrates showed an exceptional and consistent electrode–skin interfacial impedance behavior with considerably low impedance values (~4.0 × 10^5^ Ω in the Me-rich zone) and reduced variability (~2.0 × 10^6^ Ω), both very similar to what was observed for the hydrogel-based Ag/AgCl electrodes (mean 1.6 × 10^5^ Ω, variability of 6.3 × 10^5^ Ω) (see Figure 7d). The impedance variability generally was lower in the intermetallic and in the Au-rich zones. It seems that the dense, featureless morphologies with the formation of Au-rich crystalline phases exhibited by those films guarantee the electrical conductivity even under a mechanical solicitation of the PU base over time [27]. These properties led to a very distinct behavior of the Ti–Au films when applied in electrode–skin contacts for biosensing purposes.

The overall results obtained with the Ti–Me dry electrodes tested in 20 volunteers with direct skin contact under corrosive environments for 14 days confirmed the quality of the electrode–skin contact, the long-term stability, and the reusability of the prepared nanomaterials for biopotential sensing.

The development of dry electrodes based on nanostructured materials has proven to be a meaningful alternative to the standard gel-based electrodes. Flexible dry Ti–Me electrodes have been shown to overcome some of the most important limitations of gel electrodes by providing increased life span, reusability, reduced skin preparation effort, and using biocompatible Ti-based alloys applied in an increasing number of medical applications. In the study at hand, we focused on a comprehensive analysis of the electrode characteristics including chemical composition, structural composition, morphology, and the electrode–skin interface. In future studies we will compare the Ti–Me electrodes to commercially available gel-based and dry electrodes, investigating signal characteristics and signal-to-noise ratio (SNR) within ecologic biopotential monitoring applications. This will enable us to identify the optimal dry electrode materials and shapes for use in outpatient care applications. The easy and rapid dry electrode application contributes to new approaches for diagnostics and rehabilitation. Mobile, dry electrode-based biopotential monitoring devices enable person-centered continuous support and can be applied ubiquitously, without the need for trained health caregivers.

## 4. Conclusions

In this study, Ti–Me intermetallic thin films with different Me/Ti atomic ratios were deposited on different substrates to investigate their potential use as dry electrodes for biopotential sensing. Given the importance of avoiding distortions of the biopotential passing through different media, the skin–electrode interface was assessed for all the prepared electrodes. The use of nanostructured Ti–Au thin films for dry electrodes, or in specific circumstances, the use of Ti–Ag and Ti–Cu films has shown several technological advantages, including:

(i) Increased biocompatibility according to literature when compared to Ag/AgCl electrodes;
(ii) Impedance values in the same order of magnitude as the Ag/AgCl reference electrodes;
(iii) Reusability and long durability, even after repetitive applications;
(iv) Reduced variability of the impedance values recorded;
(v) Reduced costs of material and production, capable of being equitably used in new fields of application for reusable electrodes in mobile electrophysiological monitoring.

The obtained results indicate that Ti–Me thin films can be used as high-fidelity biopotential electrodes with low and stable electrode–skin impedance and long-term stable contact. Moreover, the reproducibility of the contact impedance values recorded and the reusability of the electrodes were assessed by their use over 14 days of testing on 20 different volunteers. All the Ti–Me electrodes, with both substrates (SS and PU), showed in general similar and balanced impedance between electrode sites. However, some important differences were found related to the Me selected for the Ti–Me metallic binary system and the microstructural zone where the thin films were prepared. The growing inclusion of the Me element into the Ti matrix considerably reduced the contact impedance of the electrodes and their capacitive behavior for Faradaic currents, especially evident on PU substrates. However, the Ti–Al electrodes were revealed to be prone to wider dispersions of contact impedance, especially on the flexible PU bases, most likely because of the columnar morphology and the brittle characteristics of the Ti–Al intermetallic phases structurally identified for Me/Ti ratios higher than 0.20. Conversely, the glassy structures and dense morphologies richer in Au-phases found in the films prepared with Au/Ti ratios > 1 most probably justify the stable and reproducible impedance contacts.

Developing accuracies close to the Ag/AgCl reference electrodes, the Ti–Au electrodes showed excellent performances to ensure signal quality and reliability during biopotential monitoring. In future, the electrodes developed in this work will be tested for biopotential monitoring during further in-vivo tests, to compare signal quality parameters such as the power spectral density, and the SNR.

## Data Availability

Raw data and analysis scripts are available upon request to the corresponding author.

## Sample Availability

Samples of the electrodes are available from the authors.
